# TROPOMI NO_2_ in the United States: A Detailed Look at the Annual Averages, Weekly Cycles, Effects of Temperature, and Correlation With Surface NO_2_ Concentrations

**DOI:** 10.1029/2020EF001665

**Published:** 2021-04-02

**Authors:** Daniel L. Goldberg, Susan C. Anenberg, Gaige Hunter Kerr, Arash Mohegh, Zifeng Lu, David G. Streets

**Affiliations:** ^1^ Department of Environmental and Occupational Health George Washington University Washington DC USA; ^2^ Energy Systems Division Argonne National Laboratory Argonne IL USA

**Keywords:** NO_2_ exposures, NO_2_ vs. PM2.5, NO_x_ emissions, remote sensing, TROPOMI NO_2_, weekday‐weekend effect

## Abstract

Observing the spatial heterogeneities of NO_2_ air pollution is an important first step in quantifying NO_X_ emissions and exposures. This study investigates the capabilities of the Tropospheric Monitoring Instrument (TROPOMI) in observing the spatial and temporal patterns of NO_2_ pollution in the continental United States. The unprecedented sensitivity of the sensor can differentiate the fine‐scale spatial heterogeneities in urban areas, such as emissions related to airport/shipping operations and high traffic, and the relatively small emission sources in rural areas, such as power plants and mining operations. We then examine NO_2_ columns by day‐of‐the‐week and find that Saturday and Sunday concentrations are 16% and 24% lower respectively, than during weekdays. We also analyze the correlation of daily maximum 2‐m temperatures and NO_2_ column amounts and find that NO_2_ is larger on the hottest days (>32°C) as compared to warm days (26°C–32°C), which is in contrast to a general decrease in NO_2_ with increasing temperature at moderate temperatures. Finally, we demonstrate that a linear regression fit of 2019 annual TROPOMI NO_2_ data to annual surface‐level concentrations yields relatively strong correlation (*R* ^2^ = 0.66). These new developments make TROPOMI NO_2_ satellite data advantageous for policymakers and public health officials, who request information at high spatial resolution and short timescales, in order to assess, devise, and evaluate regulations.

## Key Points

- The high instrument sensitivity of Tropospheric Monitoring Instrument (TROPOMI) can measure NO_2_ pollution with unprecedented clarity compared to predecessor instruments
- We can now quantify pollution hotspots within cities such as those related to airport/shipping operations and high traffic areas
- Annual column NO_2_ observed by TROPOMI has good correlation (*R* ^2^ = 0.66) with EPA surface observations without any surface‐to‐column conversion

## 1 Introduction

Enhancements of NO_2_ serve as a stark reminder of our society's global reliance on fossil‐fuel combustion. NO_2_—which comprises ∼70% of NO_X_ (NO_X_ = NO + NO_2_) in urban airsheds (Valin et al., 2013)—primarily originates as a byproduct of fossil‐fuel combustion, although there are some biogenic sources of NO_2_ such as lightning and microbes in soil (Jacob, 2000). NO_2_ is a toxic air pollutant, which can cause and exacerbate asthma in vulnerable populations (Achakulwisut et al., 2019; Anenberg et al., 2018) and lead to premature mortality (Burnett et al., 2004). NO_2_ can also react in the atmosphere to create tropospheric ozone (O_3_), which is noted for its damaging effects including premature aging of lungs (Broeckaert et al., 1999; McConnell et al., 2002) and premature mortality (Bell, 2004; Bell et al., 2006). HNO_3_ often represents the final chemical state of NO_2_ in the atmosphere and when deposited, agitates the equilibrium of our ecosystems due to its acidic properties (Burns et al., 2016). NO_2_ can also participate in a series of reactions to create particulate nitrate (NO_3_^−^), a component of fine particulate matter less than 2.5 microns in diameter (PM_2.5_), which is the leading cause of mortality due to air pollution (Cohen et al., 2017).

There is a rich legacy of monitoring NO_2_ by remote sensing instruments (Burrows et al., 1999). NO_2_ can be observed from space because it has unique high‐frequency spectral features within the 400–500 nm wavelength region (Vandaele et al., 1998). The newest remote sensing spectrometer, Tropospheric Monitoring Instrument (TROPOMI) (VanGeffen et al., 2019; Veefkind et al., 2012), has been gathering data on the global heterogeneities of NO_2_ air pollution since October 2017. This instrument builds on the legacy of prior Ultraviolet – Visible (UV‐Vis) spectrometers including the Global Ozone Monitoring Experiment (GOME) (Burrows et al., 1999; Martin et al., 2002; Richter & Burrows, 2002), the Scanning Imaging Spectrometer for Atmospheric Chartography (SCIAMACHY) (Bovensmann et al., 1999; Heue et al., 2005), the Global Ozone Monitoring Experiment ‐ 2 (GOME‐2) instrument (Munro et al., 2016; Richter et al., 2011), and the Ozone Monitoring Instrument (OMI) (Boersma, Eskes, Richter, et al., 2018; Krotkov, Lamsal, et al., 2017; Levelt, Oord, et al., 2006, Levelt, Joiner, et al., 2018).

Satellite‐based remote sensing instruments can be particularly useful in quantifying the trends of NO_X_ pollution in high‐emission areas (Castellanos & Boersma, 2012; Duncan et al., 2016; Georgoulias et al., 2019; Krotkov, McLinden, et al., 2016; McLinden et al., 2016; Stavrakou, Müller, Boersma, et al., 2008; Van Der A et al., 2008), the seasonal cycles of air pollution (Ialongo, Herman et al, 2016; Shah et al., 2020), and the weekly cycle of NO_X_ emissions (Beirle, Platt, et al., 2003; de Foy, Lu, & Streets, 2016; Ialongo, Herman et al, 2016; Ma et al., 2013; Russell, Valin, et al., 2010; Stavrakou, Müller, Bauwens, et al., 2020; Valin et al., 2014). In an additional step, NO_X_ emissions can be computed by combining the satellite data with meteorological information (Beirle, Borger, et al., 2019, Beirle, Boersma, et al., 2011; de Foy, Lu, Streets, Lamsal, & Duncan, 2015; Goldberg, Lu, Streets, et al., 2019; Goldberg, Saide, et al., 2019; Lorente, Boersma, et al., 2019; Lu et al., 2015; Valin et al., 2013) or by combining the satellite data with chemical transport models (Canty et al., 2015; Cooper, Martin, Padmanabhan, & Henze, 2017; Elissavet Koukouli et al., 2018; Mijling & Van Der A, 2012; Qu et al., 2017; Souri et al., 2016). Due to the consistency and robustness of the remotely sensed NO_2_ data record, scientists are beginning to infer information from the NO_2_ data about other trace gases such as CO_2_ (Goldberg, Lu, Oda, et al., 2019; Konovalov et al., 2016; Reuter et al., 2019), CH_4_ (de Gouw et al., 2020), and CO (Lama et al., 2020), since remotely sensed measurements of those trace gases are generally less reliable. Therefore, remotely sensed NO_2_ can also be helpful in indirectly estimating greenhouse gas emissions.

TROPOMI's smallest pixel size (3.5 × 7.2 km^2^ at nadir, reduced to 3.5 × 5.6 km^2^ at nadir on 6 August 2019) and enhanced sensitivity are significant improvements when compared to previous satellite instruments (Veefkind et al., 2012). NO_2_ is unique due to its relatively short photochemical lifetime which varies from 2 to 5 h during the summer daytime (Beirle, Boersma, et al., 2011; de Foy, B., Wilkins, J. L., et al., 2014; Laughner & Cohen, 2019; Valin et al., 2013) to 12–24 h during winter (Shah et al., 2020). As a result, tropospheric NO_2_ concentrations are strongly correlated with local NO_X_ emissions, which are often anthropogenic in origin.

Initial NO_2_ measurements from TROPOMI show the complex spatial heterogeneities of NO_2_ pollution with more refined resolution than any instrument before it (Griffin et al., 2019; Ialongo, Virta, et al., 2020). In particular, the smaller pixel sizes aid researchers in differentiating pollution sources within a single metropolitan area such as isolating signals from airports and individual highways (Judd, Al‐Saadi, Janz, et al., 2019). These small‐scale pixel sizes also show better agreement with the spatial features suggested by ground‐based measurements (Ialongo, Virta, et al., 2020; Judd, Al‐Saadi, Janz, et al., 2019). In particular, modeling studies have shown that matching the city‐wide NO_2_ column to 10% accuracy requires a spatial resolution of at least 4 km (Valin, Russell, Hudman, & Cohen, 2011)—the approximate spatial resolution of TROPOMI. Robust high‐spatial resolution estimates are also critical inputs to those trying to quantify the surface‐level NO_2_ exposures (Geddes, Martin, et al., 2016; Lamsal et al., 2008; Larkin et al., 2017).

The improved spatial resolution and instrument sensitivity also allow for shorter temporal averaging ranges (days to months) to gain the similar spatial structure it would normally take >1 year to gather (Beirle, Borger, et al., 2019; Dix et al., 2020; Goldberg, Lu, Streets, et al., 2019; Lorente, Boersma, et al., 2019). As a result, it is easier to gain insight on the short‐term variations of NO_X_ pollution when using TROPOMI, which can be especially helpful for those trying to quantify intra‐annual changes in NO_X_ emissions (F. Liu et al., 2020).

In this paper, we exploit TROPOMI's small pixel sizes and enhanced instrument sensitivity to analyze spatial and temporal features of NO_X_ columns in the continental United States on annual, seasonal, weekly, and daily timescales. For example, using only a short temporal range of data, we can now answer such questions as:

- Which location within each U.S. state has the worst NO_2_ air pollution?
- How does the NO_X_ emissions cycle vary by day of the week?
- How does temperature affect column NO_2_ amounts?
- How well can we infer surface‐level concentrations from satellite data?

While older sensors (e.g., OMI) provided insight into some of these questions, early sensors lacked the same sensitivity and required longer oversampling times. Therefore, answers illuminated by TROPOMI provide a “clarity” that has not been seen before.

## 2 Methods

### 2.1 TROPOMI NO_2_

TROPOMI was launched by the European Space Agency for the European Union's Copernicus Sentinel 5 Precursor (S5p) satellite mission on 13 October 2017. The satellite follows a sun‐synchronous, low‐earth (825 km) orbit with an equator overpass time of approximately 13:30 local solar time (Veefkind et al., 2012). TROPOMI measures total column amounts of several trace gases in the Ultraviolet‐Visible‐Near Infrared‐Shortwave Infrared spectral regions (VanGeffen et al., 2019). This instrument is characterized as a passive optical satellite sensor due to its reliance on solar UV‐Visible radiation to gather measurements. At nadir, pixel sizes are 3.5 × 7 km^2^ (reduced to 3.5 × 5.6 km^2^ on 6 August 2019) with little variation in pixel sizes across the 2,600 km swath. The instrument observes the swath approximately once every second and orbits the Earth in about 100 min, resulting in daily global coverage.

Using a differential optical absorption spectroscopy technique on the radiance measurements in the 405–465 nm spectral window, the top‐of‐atmosphere spectral radiances can be converted into slant column amounts of NO_2_ between the sensor and the Earth's surface (van Geffen et al., 2020). In two additional steps, the slant column quantity can be converted into a tropospheric vertical column content. In the first step, the stratospheric portion of the column (the amount above approximately 12 km in altitude) is subtracted using a global model estimate that is refined using data assimilation (Boersma, Eskes, & Brinksma, 2004). In a second step, the slant tropospheric column is converted to a vertical column using a quantity known as the air mass factor (AMF). The AMF is the most uncertain quantity in the retrieval algorithm (Lorente, Folkert Boersma, et al., 2017) and is a function of the surface reflectance, the NO_2_ vertical profile, and scattering in the atmosphere among other factors. Using accurate and high‐resolution data (spatially and temporally) as inputs in calculating the AMF can significantly reduce the overall errors of the AMF (S. Choi et al., 2019; Goldberg et al., 2017; Lamsal, 2020; Laughner, Zare, & Cohen, 2016, Laughner, Zhu, & Cohen, 2019; Lin et al., 2015; M. Liu et al., 2019; Russell, Perring, et al., 2011; Zhao et al., 2020) and thus the tropospheric vertical column content.

Operationally, the TM5‐MP model (1 × 1° resolution) is used to provide the NO_2_ vertical shape profile, and the climatological Lambertian Equivalent Reflectivity (0.5 × 0.5° resolution) (Kleipool et al., 2008) is used to provide the surface reflectivities. The operational AMF calculation does not explicitly account for aerosol absorption or scattering effects, which are partially accounted for in the effective cloud radiance fraction (Chimot et al., 2016). There is already some evidence that the current TROPOMI operational NO_2_ product may have a low bias of 20%–40% in urban areas; much of this bias may be attributed to the AMF (Judd, Al‐Saadi, Szykman, et al. 2020; Verhoelst et al., 2020). While the operational product does have larger uncertainties in the tropospheric column contents than a product with higher spatial resolution inputs, we limit our analysis to relative trends, which dramatically reduces this uncertainty.

### 2.2 Re‐gridding

For our analysis we re‐grid the operational TROPOMI tropospheric vertical column NO_2_, with native pixels of approximately 3.5 × 7 km^2^, to a newly defined 0.01° × 0.01° grid (approximately 1 × 1 km^2^) centered over the continental United States (CONUS; corner points: SW: 24.5°N, 124.75°W; NE: 49.5°N, 66.75°W). Before re‐gridding, the data are filtered so as to use only the highest quality measurements (quality assurance flag (QA_flag) > 0.75). By restricting to this QA value, we are removing mostly cloudy scenes (cloud radiance fraction > 0.5) and observations over snow‐ice. Once the re‐gridding has been completed, the data are averaged over varying timeframes as discussed in the results section.

### 2.3 Other Data sets

Additionally, we use three complementary products in some sections of our analysis. We compare tropospheric vertical column information from TROPOMI to the same quantity from the NASA OMI NO_2_ version 4 product in a qualitative sense. Only OMI pixels with cloud fractions < 0.3, surface albedo < 0.3, and not affected by the “row anomaly” are included. When filtering TROPOMI data based on temperature, we use the maximum daily hourly 2‐m temperature from the ERA5 re‐analysis. To downscale the ERA5 re‐analysis, which is provided at 0.25° × 0.25°, we spatially interpolate maximum daily hourly 2‐m temperature to 0.01° × 0.01° using bilinear interpolation. For that reason, the heat‐urban island effect and any microscale meteorology features (e.g., sea breezes) will not be accounted for, but these effects should be minor for our particular analysis, which groups temperatures in ∼5°C intervals.

## 3 Results

### 3.1 TROPOMI NO_2_ in CONUS

**Figure 1:**
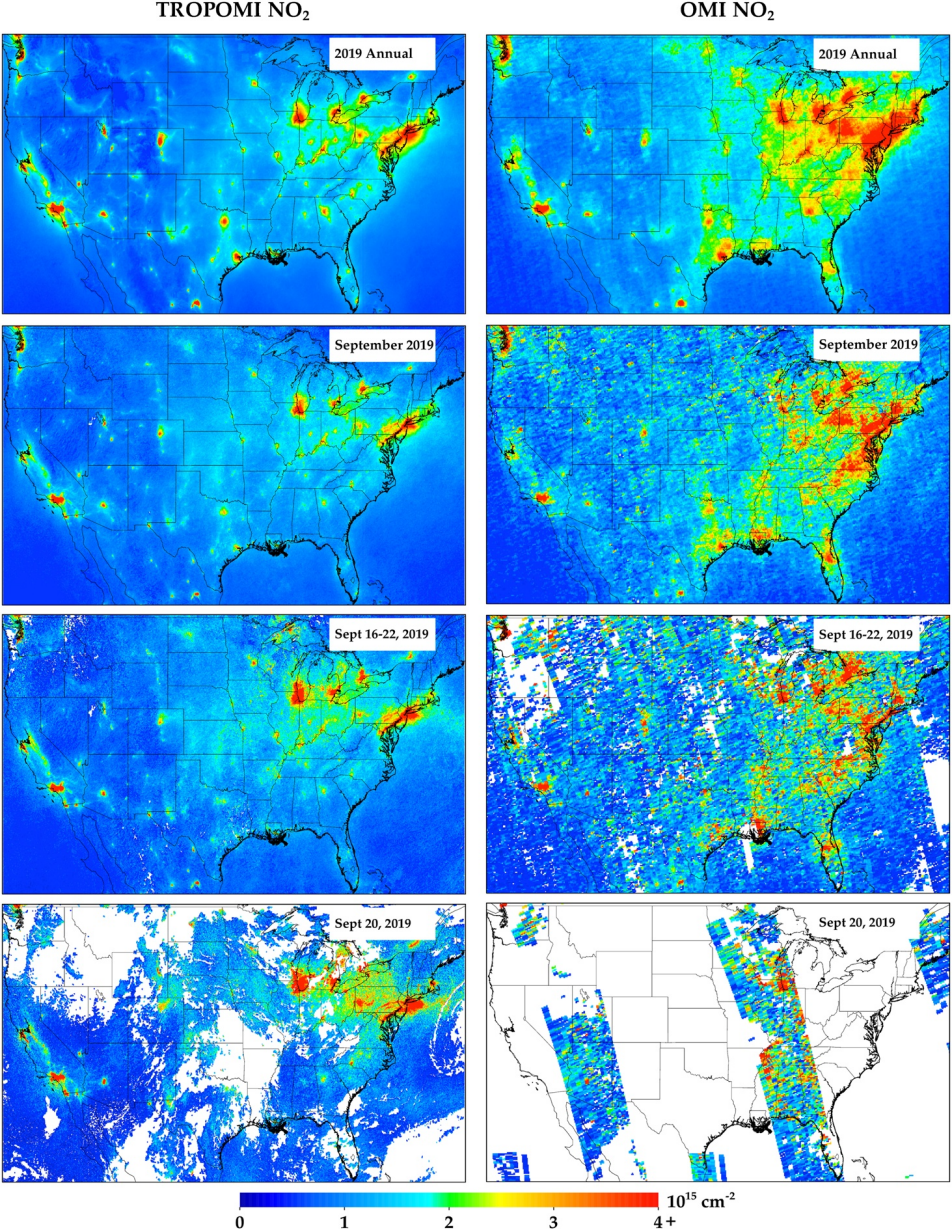
(Left) TROPOMI NO_2_ and (right) OMI NO_2_ oversampled to 0.01° × 0.01° spatial resolution for four different temporal resolutions: (top row) annual, (second row) monthly, (third row) weekly, and (bottom row) daily.

Figure 1 depicts the 2019 CONUS annual average of TROPOMI and OMI tropospheric vertical column NO_2_ compared to averages over monthly, weekly, and daily timeframes.

This example illustrates how shorter timeframes compare to the annual average in both magnitude and clarity. In the single daily snapshot (20 September 2019), there are wide sections that are missing due to cloud coverage. Missing data in the OMI NO_2_ snapshot is much more widespread than TROPOMI due to the “row anomaly.” which obstructs a portion of OMI's field of view. In the areas that do have coverage, values can be a factor of five different than the annual average, but the spatial heterogeneities are generally captured. When oversampling over a one‐week period (16–22 September 2019), the TROPOMI image quickly starts to resemble the annual average with some differences in magnitude due to meteorological factors, such as temperature (which will be discussed later), but the OMI image is still very noisy. A monthly oversampled image essentially captures the same spatial heterogeneities as the annual average, but with magnitude differences due to meteorology. In most scenarios, a one‐month average should be considered the minimum amount of oversampling time needed for TROPOMI to properly capture spatial heterogeneities, while for OMI ∼12 months of data is needed in order to properly capture spatial heterogeneities. It should be noted that September was specifically chosen for this analysis due to its propensity to have both less cloud coverage and snow cover than other months. If oversampling during winter months (i.e., December–March), which tend to have fewer ideal conditions for satellite retrievals of trace gases, oversampling times will need to be longer to achieve similar clarity. In a qualitative sense, OMI yields larger values than TROPOMI in most areas (rural and urban alike). This is consistent with other literature, which shows OMI yielding larger values than TROPOMI (Wang et al., 2020) and a low bias in TROPOMI in U.S. urban areas (Judd, Al‐Saadi, Szykman, et al., 2020).

**Figure 2:**
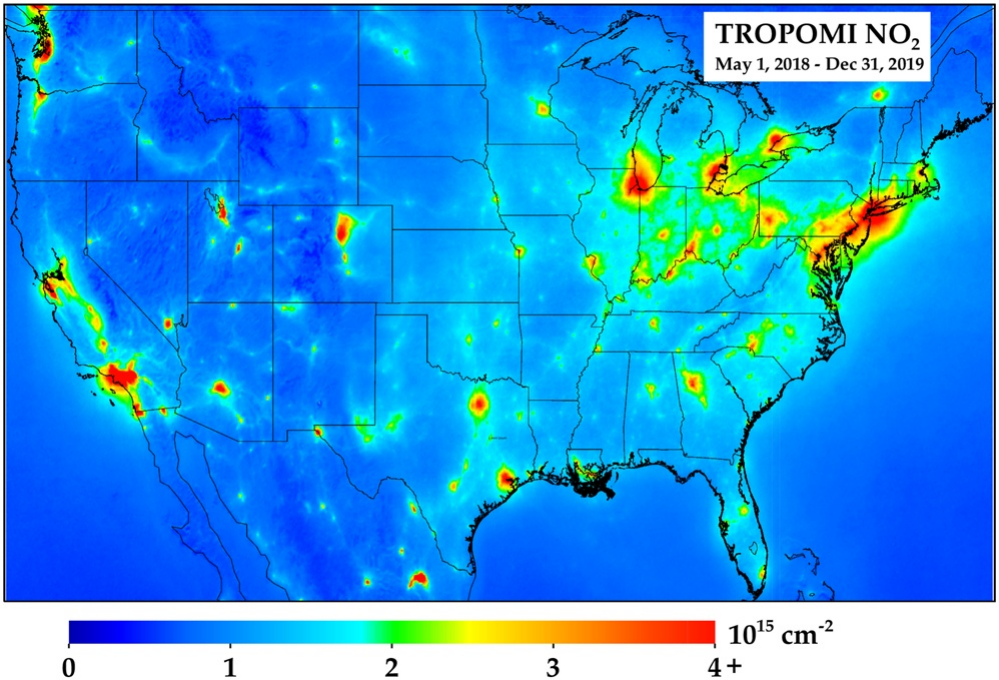
TROPOMI NO_2_ oversampled to 0.01° × 0.01° spatial resolution during 1 May 2018–31 December 2019. Only pixels exceeding a quality assurance flag of 0.75 are included.

When visually inspecting the CONUS TROPOMI NO_2_ average during the initial 20 months of the TROPOMI record (1 May 2018–31 Dec 2019) (Figure 2), we now start to see clear spatial heterogeneities across the domain. The largest U.S. cities can be seen, and their magnitudes can be compared to each other (results further discussed later).

**Figure 3:**
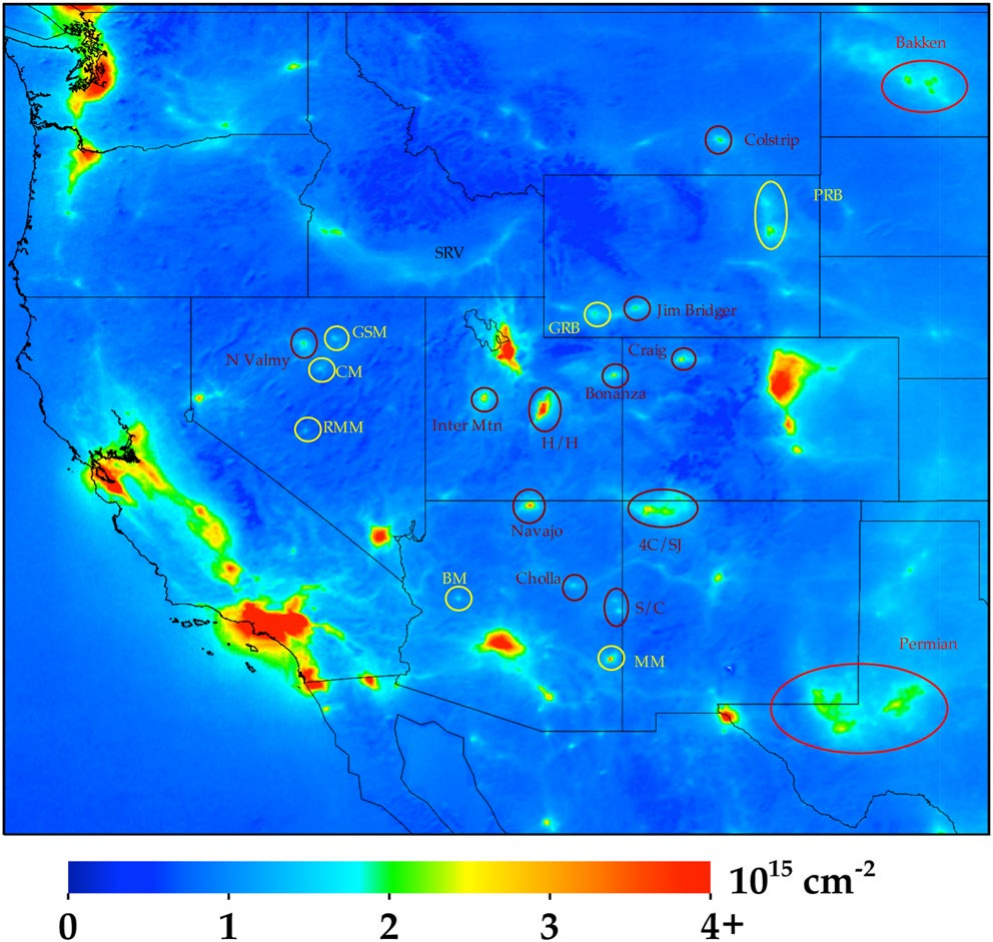
Same data shown in Figure 2, but now zoomed into the western United States. Power plants are outlined in dark magenta, mining operations in yellow, and oil & gas in bright red.

Equally important, smaller sources of NO_2_ pollution can now be observed, and they are not spatially smeared into the background NO_2_ concentration. For example, when magnifying the western United States (Figure 3), the roadway network and related activity in the Idaho Snake River valley can be clearly observed. Other examples are the copper mining operations in Arizona associated with the Morenci Mine and Bagdad Mine, the coal mining operations in the Powder River Basin and Green River Basin in Wyoming, and to a lesser extent the gold mining operations associated with the Goldstrike, Cortez, and Round Mountain mines in Nevada. In addition, NO_2_ concentrations are clearly correlated with oil & gas operations in the Permian (Texas) and Bakken (North Dakota) basins (also discussed in Dix et al., 2020) and are > 5 times larger than the NO_2_ in the rural areas upwind. Individual spikes in NO_2_ associated with NO_X_ emissions from large power plants (e.g., Navajo, Cholla, Springerville/Coronado (S/C) in Arizona, Craig in Colorado, Colstrip in Montana, N Valmy in Nevada, Four Corners/San Juan (4C/SJ) in New Mexico, Intermountain, Bonanza, Hunter/Huntington (H/H) in Utah, Jim Bridger in Wyoming) can also be observed during this 2018–2019 period even though there have been large reductions (∼85%) in the NO_X_ emissions from most of these power plants since the introduction of the federally mandated NO_X_ SIP call in 2003.

**Figure 4:**
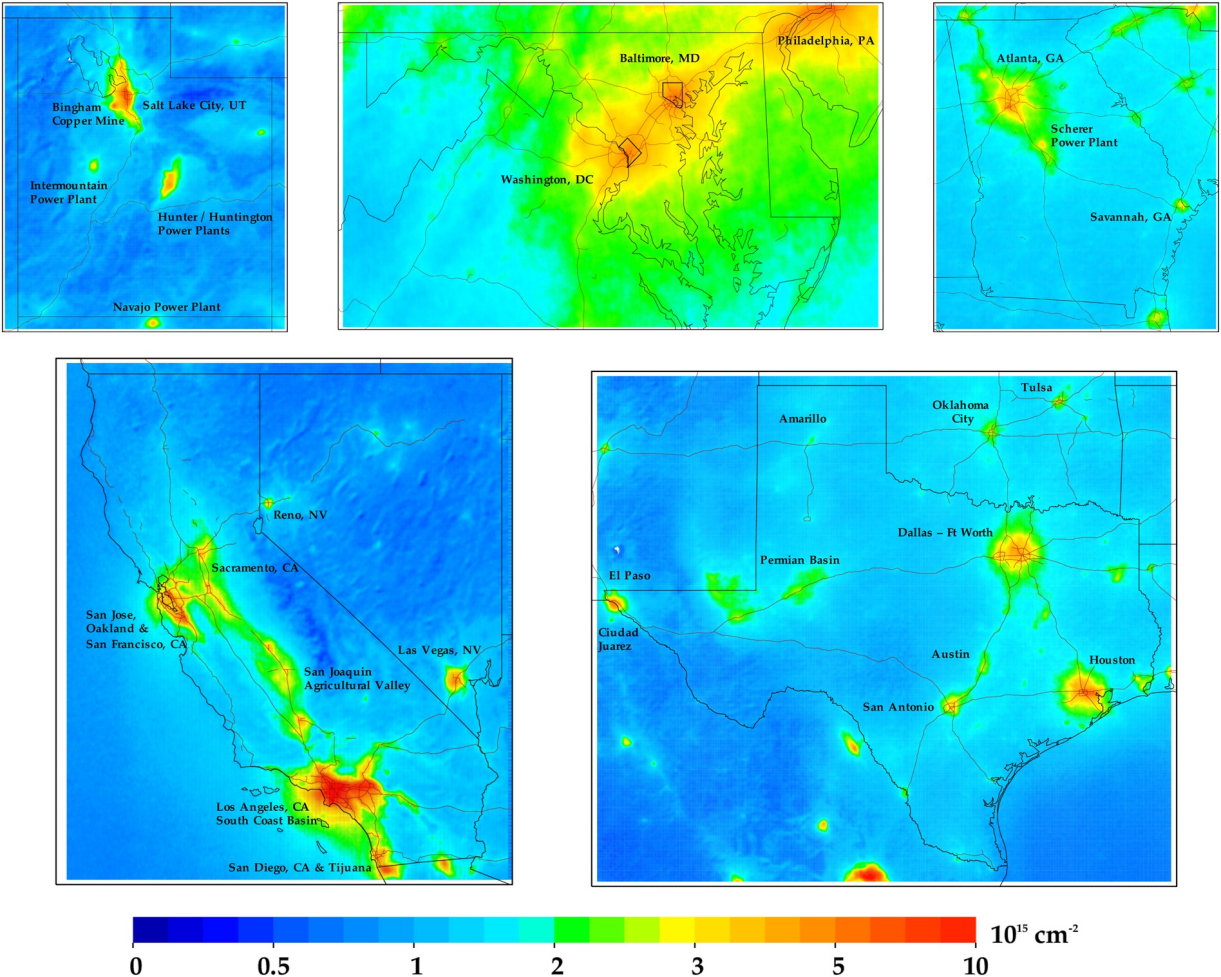
Same data shown in Figure 2, but now zoomed into five different U.S. states. Color bar has been adjusted to better differentiate spatial heterogeneities on a local scale.

TROPOMI data are especially powerful in analyzing local variations in NO_2_ pollution as compared to predecessor instruments. In Figure 4, we zoom into five different U.S. states, and in Table 1 we provide the largest NO_2_ values in each state; note that in Figure 4 we use a colorbar that is not linear in order to better differentiate urban versus rural values.

**Table 1:** *Largest NO*_*2*_ *Column Value in Each U.S. State During the 1 May 2018–31 Dec 2019 Period*

U.S. state	Latitude (°N)	Longitude (°E)	NO2 (molec/cm2)	Detailed location
CA	34.03	−118.18	1.41E+16	E Los Angeles, CA
NY	40.72	−73.97	1.13E+16	East River, Brooklyn, NY
NJ	40.69	−74.14	9.75E+15	Port Newark, NJ
IL	41.82	−87.77	7.31E+15	Cicero, Chicago, IL (near MDW)
WA	47.46	−122.26	6.90E+15	Tukwila, WA (SE Seatle)
IN	41.66	−87.47	6.28E+15	E Chicago, IN (Steel Mill)
UT	40.71	−111.9	6.18E+15	S Salt Lake City, UT
CO	39.76	−105.02	5.98E+15	Highland, Denver, CO
PA	39.95	−75.16	5.95E+15	Downtown Philadelphia, PA
AZ	33.47	−112.15	5.87E+15	Cuatro Palmas, Phoenix, AZ
MI	42.31	−83.11	5.74E+15	Detroit, MI
TX	29.74	−95.14	5.58E+15	Deer Park, Houston, TX
CT	41	−73.67	5.46E+15	Greenwich, CT
NV	36.1	−115.18	4.97E+15	Las Vegas Strip, Las Vegas, NV
MD	39.28	−76.6	4.94E+15	Port of Baltimore, Baltimore, MD
DC	38.89	−77.01	4.65E+15	Capitol Hill, Washington, DC
GA	33.64	−84.42	4.65E+15	Hartsfield Airport, Atlanta, GA
VA	38.88	−77.05	4.59E+15	Pentagon, Arlington, VA
DE	39.8	−75.37	4.34E+15	Claymont, Wilmington, DE
OR	45.52	−122.65	4.25E+15	Buckman, Portland, OR
KY	38.18	−85.73	4.21E+15	Louisville, KY (Airport)
OH	39.12	−84.54	4.20E+15	Cincinnati, OH
MA	42.37	−71.06	4.14E+15	Charlestown, Boston, MA (near BOS)
LA	29.93	−90.14	3.98E+15	Mississippi River, New Orleans, LA
NC	35.24	−80.85	3.76E+15	Catawba, NC (near Marshall Steam PP)
WV	38.94	−82.11	3.68E+15	Lakin, WV (near Gavin PP)
MO	38.68	−90.19	3.67E+15	Mississippi River, St Louis, MO
KS	39.12	−94.6	3.61E+15	Missouri River, Kansas City, KS
TN	36.16	−86.77	3.52E+15	Nashville, TN
FL	25.85	−80.34	3.40E+15	Medley, Miami, FL
WI	42.86	−87.82	3.40E+15	Oak Creek, WI (near Oak Creek PP)
MN	44.97	−93.24	3.28E+15	Mississippi River, Minneapolis, MN
AL	33.52	−86.82	3.21E+15	Fountain Heights, Birmingham, AL
RI	41.8	−71.41	2.88E+15	S Providence, RI
IA	41.25	−95.88	2.79E+15	Council Bluffs, IA
NE	41.25	−95.88	2.79E+15	Missouri River, Omaha, NE
OK	36.16	−96	2.64E+15	Tulsa, OK
WY	43.69	−105.32	2.52E+15	Thunder Basin Coal, WY
SC	32.88	−79.99	2.52E+15	N Charleston, SC
NM	35.11	−106.62	2.51E+15	Albuquerque, NM
AR	35.12	−90.1	2.46E+15	W Memphis, AR
ID	43.58	−116.23	2.30E+15	Boise, ID (Airport)
ND	47.35	−101.81	2.24E+15	Beulah, ND (near Dakota Gasification Co)
MT	45.86	−106.57	2.20E+15	Colstrip, MT (near Colstrip PP)
NH	42.94	−70.81	1.93E+15	Hampton, NH
ME	43.66	−70.29	1.90E+15	Portland, ME
MS	32.34	−90.19	1.77E+15	Jackson, MS
SD	43.6	−96.74	1.53E+15	N Sioux Falls, SD
VT	42.91	−73.18	1.49E+15	Wilmington, VT

*Note*. Ordered by largest to smallest maximum value.

**Figure 5:**
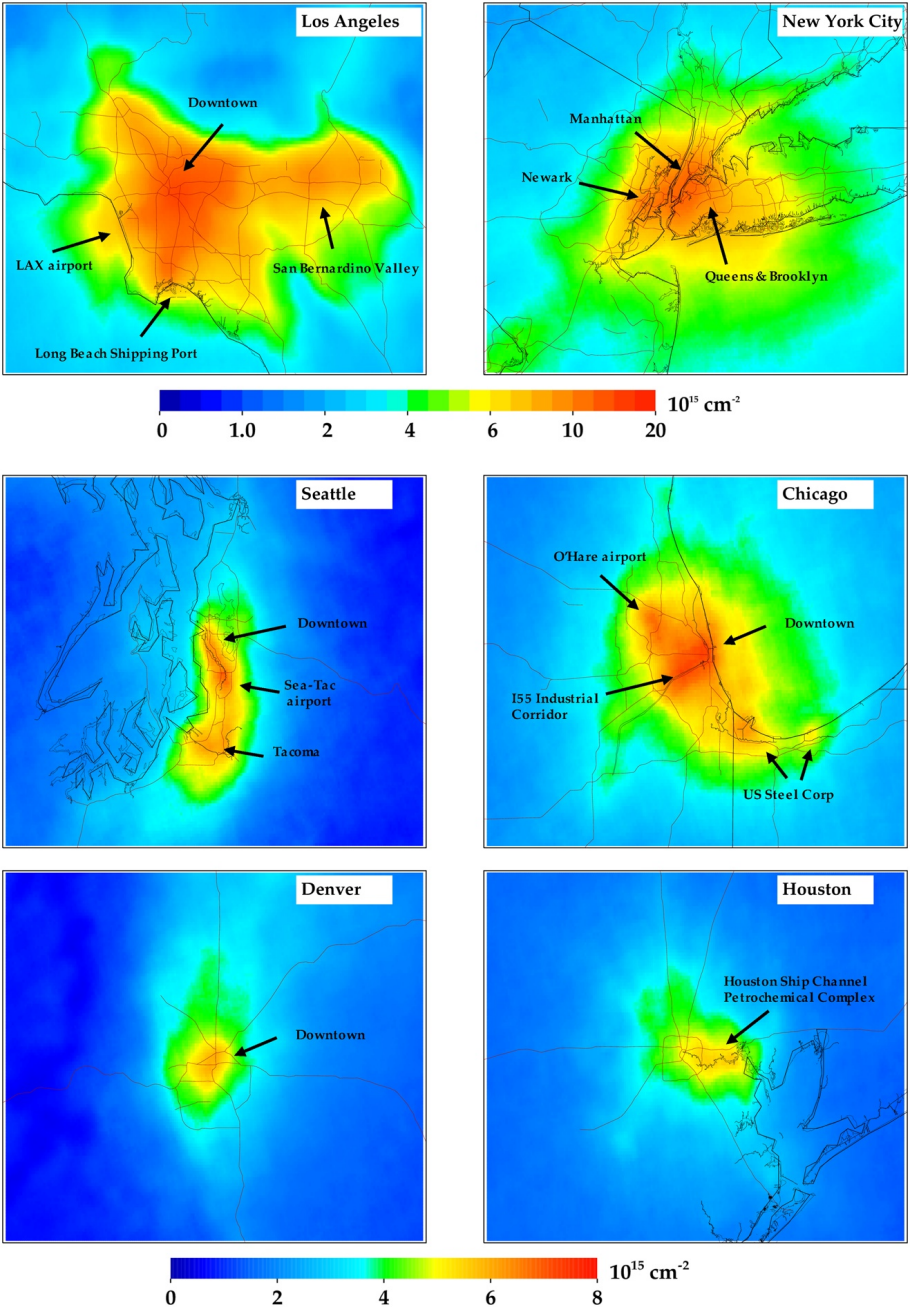
Same data shown in Figure 2, but now zoomed into six different U.S. cities. Color bar has been adjusted to better differentiate spatial heterogeneities on a local scale.

In Figure 5, we zoom into six different U.S. cities. In each instance, the oversampled TROPOMI NO_2_ images exhibit features that match known NO_X_ emissions patterns. The larger NO_2_ values correlate very well to the interstate network, population density, and industrial activity hubs (such as manufacturing facilities, airports, and shipping ports). For example, in the image of Maryland, the largest value is observed at the Baltimore Harbor, which is a confluence of several major highways, a large shipping port, the city incinerator, and many industrial facilities. Similarly, the largest values in Chicago exist along the I‐55 corridor which has a high traffic volume and a high‐density of industrial facilities, with secondary maxima at the O'Hare International airport and the U.S. Steel Corp operations in East Chicago, Indiana. In Los Angeles, the spatial pattern matches the basin outline very well, with the largest values between downtown Los Angeles and the Long Beach Shipping Port. In Houston, Texas the largest values are nearest to the petrochemical refining facilities east of town. For all cases, TROPOMI can accurately quantify the relative relationship between the largest sources of NO_X_ emissions and NO_2_ concentrations.

### 3.2 Day‐of‐the‐Week Relationships

**Figure 6:**
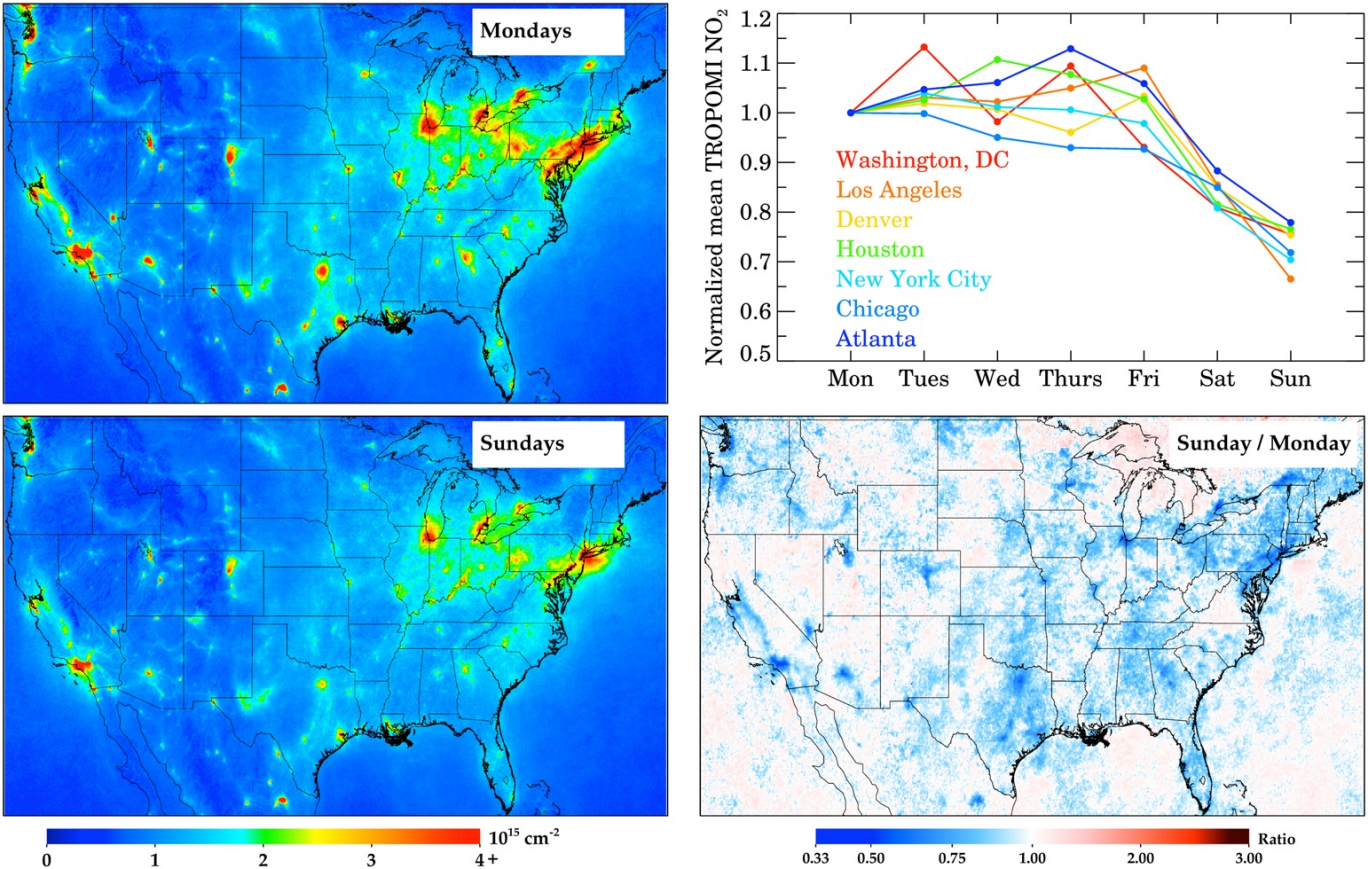
Weekly variations in column NO_2_. (Top left) TROPOMI NO_2_ during Mondays. (Bottom left) TROPOMI NO_2_ during Sundays. (Top right) Weekly variation of TROPOMI NO_2_ in seven U.S. cities normalized to Mondays; city averages are across a 1° × 1° box centered on the city. (Bottom right) Ratio between Sundays and Mondays.

A common use of oversampled satellite data is in investigating the weekly cycle of NO_X_ emissions. In Figure 6, we show the weekly pattern of NO_2_ across CONUS for three different days of the week as well as the full weekly cycle in seven U.S. cities; we selected U.S. metropolitan areas that were both large and representative of geographic diversity. In all cities, the NO_2_ appears to be approximately equivalent amongst all weekdays with some minor exceptions. NO_2_ pollution is 2.5% larger on Tuesday than a typical weekday, while Mondays and Fridays have 1.4% and 1.3% lower NO_2_ pollution than a typical weekday. On Saturdays, NO_2_ is 16% lower than the weekday averages, and on Sundays 24% lower. Standard errors of the mean for each city are shown in Table S1, and are approximately 10% for any given city, and approximately 4% when all cities are aggregated together. This means that NO_2_ changes on weekends—including the differences between Saturdays and Sundays—are statistically significant, but the difference between weekdays are not yet statistically significant. As more TROPOMI NO_2_ data are acquired over time, these standard errors of the mean will decrease, and we might be able to deduce statistically significant changes between individual weekdays. The weekend changes calculated here (16% drop on Saturdays, 24% drop on Sundays) are less dramatic than previously reported weekend changes (30%–60% drops) in the 2005–2013 timeframe (de Foy, Lu, & Streets, 2016; Russell, Valin, et al., 2010; Valin et al., 2014). There are two explanations for the flattening of the weekday‐weekend cycle: 1.) as overall emissions are decreasing, the NO_2_ lifetime in many cities is increasing (Stavrakou, Müller, Bauwens, et al., 2020) and 2.) passenger vehicles, which have a pronounced weekday‐weekend emissions pattern, are continually representing a smaller fraction of NO_X_ emissions over time (Dallmann & Harley, 2010; McDonald et al., 2012).

When analyzing the weekday/weekend differences, there should be some consideration for the difference in traffic patterns and general activity between weekends and weekdays. On weekends, traffic counts generally peak in the early afternoon, while on weekdays traffic counts peak in the evening, with a secondary peak in the early morning (de Foy, 2018). Since the satellite observation is acquired in the early afternoon, we suggest that the 24‐h averaged NO_X_ emissions difference between weekdays and weekends may be even greater than implied by the satellite data. The soon‐to‐be‐launched TEMPO instrument, a geostationary satellite, will hopefully be able to better quantify the morning and evening differences of NO_X_ emissions (Chance et al., 2019; Penn & Holloway, 2020; Zoogman et al., 2017).

### 3.3 Hot Versus Warm Days

**Figure 7:**
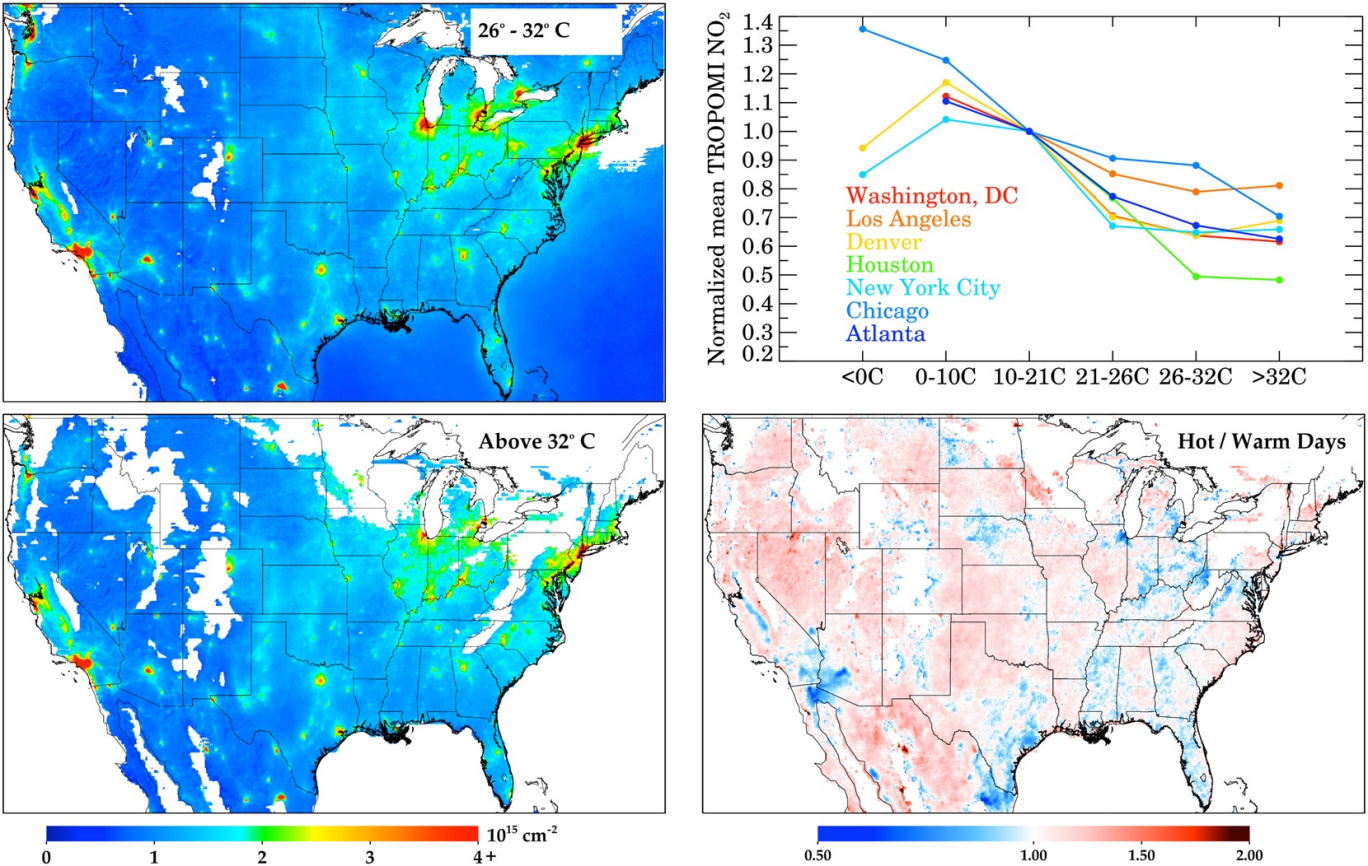
Temperature variations in column NO_2_. (Top left) TROPOMI NO_2_ when maximum daily 2‐m temperature is between 26°C–32°C (Warm; 80°F–90°F); only areas where >10 valid pixels are shown. (Bottom left) TROPOMI NO_2_ when maximum daily 2‐m temperature is greater than 32°C (hot; 90°F); only areas where >10 valid pixels are shown. (Top right) Temperature variation of TROPOMI NO_2_ in seven U.S. cities normalized to 10°C–21°C (50°F–70°F); city averages are across a 1° × 1° box centered on the city. (Bottom right) Ratio between days with daily 2‐m temperature >32°C (Hot) and 26°C–32°C (Warm).

In Figure 7, we show the variation in column NO_2_ as a function of the daily maximum 2‐m temperature. Due to varying climates across the United States, most cities do not have values for all temperature bins. In general, as temperatures increase, NO_2_ decreases; this is primarily driven by j(NO_2_) which increases with stronger sunlight. When temperatures are >32°C, we observe a leveling with increasing temperature. This may be related to increasing anthropogenic NO_X_ emissions (Abel et al., 2017; He et al., 2013) at high temperatures despite a shorter NO_2_ lifetime. This may also be driven by biogenic or natural causes, such as the faster dissociation of peroxy‐acyl nitrates (PANs) or increased soil NO_X_ emissions (Rasool et al., 2019; Romer et al., 2018) at hot temperatures. The latter reasons are likely causing rural areas to observe increases in NO_2_ as temperatures warm above 32°C. The temperature‐driven stabilization of NO_2_ at very high temperatures appears to hold for all cities except Chicago. Standard errors of the mean for each city are shown in Table S2, and are approximately 7% for any given city on warm/hot temperature days (>20°C), and approximately 2%–3% when all cities are aggregated together. This means that the NO_2_ decreases with increasing temperature as well as the NO_2_ increases on the hottest days are statistically significant in most areas. It should be noted that there are cross‐correlations with increased temperature such as a lower solar zenith angle (which affects photolysis rates of chemical species and the satellite viewing geometry), larger biogenic volatile organic compound (BVOC) emissions in forested areas (which affects the NO_2_ lifetime), and higher total water columns (which affects wet deposition and introduces an increased spectral interference). Apportionment of the effects of natural versus anthropogenic sources contributing to NO_2_ increases in urban areas on the hottest days will be the subject of future research using model simulations.

### 3.4 Relationship With Surface NO_2_ Concentrations

**Figure 8:**
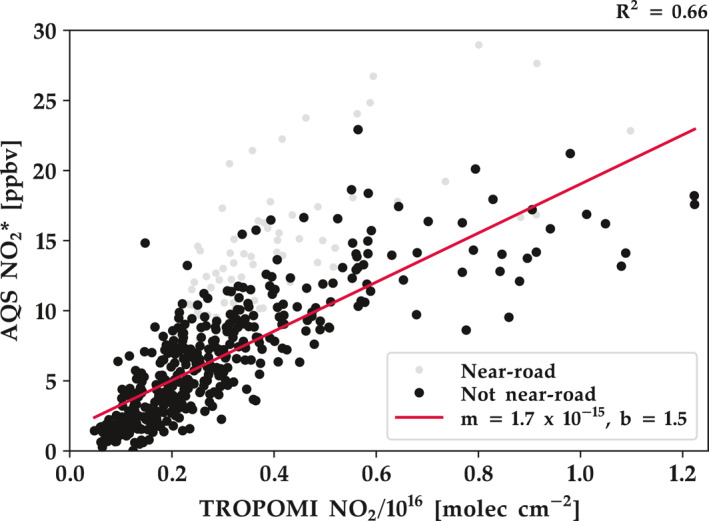
EPA AQS annual surface NO_2_* observations for 2019 compared to the collocated oversampled 0.01° × 0.01° TROPOMI value during the same timeframe. R^2^ represents the correlation between TROPOMI and not near‐road monitors.

To understand how well TROPOMI NO_2_, without any adjustment, captures surface‐level concentrations, we compare the 2019 annual TROPOMI NO_2_ average to 24‐h annual average EPA AQS monitor data. The surface‐level concentrations from the EPA AQS network are known to have a high instrument bias (Dickerson et al., 2019) and thus referred to as NO_2_* hereafter. In Figure 8, we show a scatterplot between 2019 annual averages of oversampled TROPOMI NO_2_ and AQS surface‐level NO_2_* concentrations. For our analysis, we restrict our fit to monitoring sites that are not “near‐road.” The EPA requests certain states to site “near‐road” NO_2_ monitors, which are requested to be within 20 m of a major highway; we do not expect TROPOMI observations to capture this very fine spatial gradient, and are therefore not considered in our fit. Figure 8 demonstrates that there is a strong correlation (*R* ^2^ = 0.66) between a linear fit and monitoring sites considered to be “not near‐road.” which suggests that many (but not all) of the spatial heterogeneities observed by TROPOMI over long time intervals (e.g., year) are real and not an artifact of the processing algorithms. We are encouraged to see that a simple linear fit is able to capture near‐surface NO_2_ variability well. In order to better estimate surface‐level concentrations, TROPOMI NO_2_ data should be merged with a model simulation (Cooper, Martin, McLinden, & Brook, 2020) and/or land‐use characteristics (Bechle et al., 2015; Beloconi & Vounatsou, 2020; Di et al., 2019; Larkin et al., 2017).

## 4 Conclusions

This study investigates the capabilities of the TROPOMI in observing the spatial and temporal patterns of NO_2_ pollution in the continental United States (CONUS). Here, we demonstrate that TROPOMI can capture fine‐scale spatial heterogeneities in urban areas, such as emissions related to airport/shipping operations and high traffic; this type of spatial precision cannot be matched by predecessor satellite instruments over short timescales (<1 year). We find that Saturday and Sunday concentrations are 16% and 24% lower respectively than during weekdays, with the caveat that diurnal emissions patterns vary among weekdays and weekends. We also analyze the effects of hot temperatures (>32°C) on NO_2_ column amounts and find that column NO_2_ is generally larger on the hottest days as compared to warm days (26°C–32°C). Finally, we compare column NO_2_ with surface‐level NO_2_ estimates and find relatively good correlation (*R* ^2^ = 0.66).

For this work, we rely on the operational TROPOMI NO_2_ algorithm, which underestimates tropospheric vertical column NO_2_ in urban areas. Previous studies suggest that this underestimate is due to the AMF and ∼5km pixel size which cannot resolve street‐level variations in concentrations (Goldberg, Lu, Streets, et al., 2019; Griffin et al., 2019; Judd, Al‐Saadi, Szykman, et al., 2020; Judd, Al‐Saadi, Janz, et al., 2019; Zhao et al., 2020); investigating the effects of the AMF bias on trends as well as investigating the effects of the pixels sizes will be the subject of future work. Also, there may be a clear‐sky bias (Geddes, Murphy, et al., 2012) associated with any satellite retrieval, but the general spatial heterogeneities of NO_2_ pollution should be similar amongst all types of weather conditions when averaged over long timeframes. Lastly, interpreting results from polar‐orbiting satellite instruments, such as TROPOMI, should be made with some caution due to the mid‐day only data collection time. Work quantifying this bias has shown that NO_2_ column measurements are lower and incrementally more spatially homogeneous in the afternoon than during the morning (Chong et al., 2018; Fishman et al., 2008; Herman et al., 2019; Knepp et al., 2015; Penn & Holloway, 2020; Tzortziou et al., 2015); it is likely that data from geostationary platforms such as TEMPO (Zoogman et al., 2017), GEMS (W. J. Choi, 2018), and Sentinel 4 (Timmermans et al., 2019), will be able to provide further insight on this time‐of‐day bias.

Because TROPOMI can observe and measure NO_2_ increases attributed to relatively small sources, future work should be able to quantify emissions from small sources (e.g., industrial activities, ship plumes, small wildfires) that had previously gone undetected from predecessor space‐based instruments. Furthermore, due to the instrument's excellent stability, precision, and spatial resolution, it is no longer necessary to average over 6+ months of data to gain a clear depiction of regional NO_2_ abundances; instead monthly, weekly or even daily aggregations could suffice for many purposes. The examples presented here demonstrate how TROPOMI NO_2_ satellite data can be advantageous for policymakers requesting information at high spatial resolution and short timescales, in order to assess, devise, and evaluate regulations. Future health impact assessment studies can use the high‐spatial resolution capabilities of TROPOMI NO_2_ to investigate disparities in traffic‐related air pollution exposure and associated health effects between neighborhoods and population sub‐groups within cities.

## Supporting information

Supporting Information S1

## Data Availability

TROPOMI NO_2_ data can be freely downloaded from the European Space Agency Copernicus Open Access Hub or the NASA EarthData Portal (http://doi.org/10.5270/S5P-s4ljg54). ERA5 can be freely downloaded from the Copernicus Climate Change (C3S) climate data store (CDS) (https://cds.climate.copernicus.eu/#!/search?text=ERA5&type=dataset).
