# Supplementary material for: TROPOMI NO2 in the United States: A Detailed Look at the Annual Averages, Weekly Cycles, Effects of Temperature, and Correlation With Surface NO2 Concentrations
Source: Earths Future. 2021 Apr 2;9(4):e2020EF001665. doi: 10.1029/2020EF001665 (PMC8047911; doi:10.1029/2020EF001665)
Supplement: Supplementary file 1 — Supporting Information S1 [file EFT2-9-e2020EF001665-s001.docx]

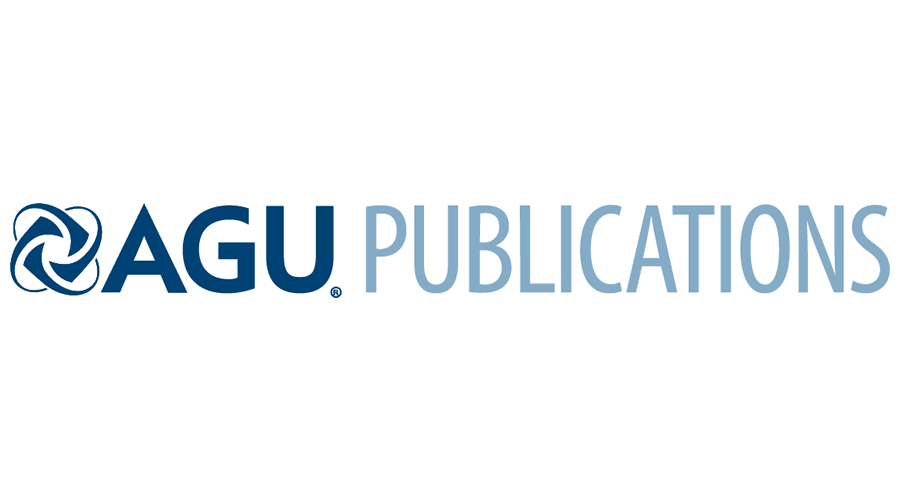


Supporting Information for:

**TROPOMI NO_2_ in the United States: A detailed look at the annual averages, weekly cycles, effects of temperature, and correlation with PM_2.5_**

Daniel L. Goldberg*^,1,2^, Susan Anenberg^1^, Gaige Hunter Kerr^1^, Arash Mohegh^1^, Zifeng Lu^2^, David G. Streets^2^

^1^Department of Environmental and Occupational Health, George Washington University, Washington, DC, U.S.

^2^Energy Systems Division, Argonne National Laboratory, Lemont, IL, U.S.

^*^Corresponding author. Phone: (202)994-8102; Email: [dgoldberg@gwu.edu](mailto:dgoldberg@gwu.edu)

**This PDF file includes:**

Table S1. Standard Relative Errors of the Means for the Weekly Cycle

Table S2. Standard Relative Errors of the Means for the Temperature Effects
